# Spotting the “small eyes”: using photo-ID methodology to study a wild population of smalleye stingrays (*Megatrygon microps)* in southern Mozambique

**DOI:** 10.7717/peerj.7110

**Published:** 2019-06-11

**Authors:** Atlantine Boggio-Pasqua, Anna L. Flam, Andrea D. Marshall

**Affiliations:** 1 Marine Megafauna Association, Tofo Beach, Inhambane, Mozambique; 2 AgroParisTech, Paris, France

**Keywords:** Smalleye stingray, *Megatrygon microps*, Photo-identification, Mozambique, Population, Habitat, Movement, Natural markings, Pattern validation, Migration

## Abstract

**Background:**

The smalleye stingray (*Megatrygon microps*) is a large and rare dasyatid ray, patchily distributed across the Indo-West Pacific. Free-swimming individuals have regularly been recorded in Southern Mozambican coastal waters utilizing different inshore environments. Distinctive features of the species include latitudinal rows of white spots on the dorsal surface of their pectoral disc.

**Methods:**

This study aimed to determine if the natural spot patterns on *M. microps* are sufficiently unique and stable to use in photo-identification studies of wild populations. Research dive logs were combined with opportunistic photographs from local dive centers and recreational divers to create a photographic database from the Inhambane Province coastline.

**Results:**

Seventy different individuals were identified over a 15-year period, all exhibiting uniquely identifiable patterns. Stingrays were easily identifiable over a period of six years with multiple re-sightings of the same individuals recorded. Analysis of encounters across the Inhambane coastline revealed that individual rays regularly use inshore reefs along a 350 km stretch of coastline. Fifteen stingrays were re-sighted during the study period, including one showing a 400 km return movement between Tofo Beach and the Bazaruto Archipelago, which is the longest distance traveled by a dasyatid ray on record. Several presumably pregnant females have also been recorded in the Bazaruto Archipelago National Park.

## Introduction

*Megatrygon microps* (*Dasyatis microps*, Annandale, 1908; *Megatrygon* in revised classification of Last, Naylor & Manjaji-Matsumoto (2016) is commonly described as the largest marine stingray in the world, reaching disc widths of up to 222 cm (Garman, 1913). In the literature *M. microps* has been reported in estuaries, river mouths, and inshore coastal waters. Very little is known about the species’ biology, behavior, range, abundance, population trends, reproduction, or threats. Consequently, smalleye stingrays remain listed as “Data Deficient” in the IUCN Red List Threatened Species (Fahmi et al., 2016). While its reported range is vast throughout the Indo-West Pacific (Purushottama et al., 2018), it is now believed that this species may be patchily distributed within this region and have fragmented populations. Historically, it has been reported in the Bay of Bengal (Kapoor, Dayal & Ponniah, 2002), the Arabian Gulf, the Arabian Sea (Osmany, Moazzam & Ayub, 2015), the Maldives, South-East Asia, and North-East Australia (Meekan et al., 2016). *M. microps* was first recorded in Mozambique in 2004 off Tofo Beach (Pierce, White & Marshall, 2008) where free-swimming individuals were encountered using the same cleaning stations as manta rays. Since then, these rays have been regularly observed by divers along the coast who have encountered them from the Bazaruto Archipelago National Park in the north of the Inhambane Province to Zavora the southernmost extent of the province.

The primary aim of this study was to assess the feasibility of photographic identification methodology to study wild populations of *M. microps*. Photographic identification or “photo-ID” refers to a methodology whereby individual animals are identified and tracked using their unique natural patterns and/or other distinct features. It has been successfully used in a variety of marine species to learn more about wild populations, including many species of rays like manta rays (Marshall, Dudgeon & Bennett, 2011), skates (Benjamins et al., 2018), and eagle rays (González-Ramos et al., 2016; Flowers et al., 2017; Cerutti-Pereyra et al., 2018). Conventional tagging studies can be invasive, at times affecting natural behaviour and individual fitness (Manire & Gruber, 1991; Fouts & Nelson, 1999; Feldheim, Gruber & Ashley, 2002; Dicken, Booth & Smale, 2006; Wilson & McMahon, 2006). They also depend on the application of temporary physical tags which can be expensive, may require surgery or if externally placed can be easily shed, removed, damaged or biofouled (Barrowman & Myers, 1996; Kohler & Turner, 2001; Feldheim, Gruber & Ashley, 2002; Dicken, Booth & Smale, 2006; Pierce & Bennett, 2009). In comparison, photo-ID methods are relatively non-invasive, rely on natural and more permanent markings, which can last over the course of an animal’s life and can be easily performed with affordable photographic equipment (Pierce et al., 2018). Photo-ID methods thus present an attractive alternative or complement to conventional tagging studies, with potential applications including the evaluation of site affinity, inter-site movements, population size, and demographics (Marshall & Pierce, 2012).

The use of photo-ID methodology is only suitable in species that (a) have distinct, long-term markings, (b) frequent areas accessible to observers, and (c) are reasonably easy to approach and photograph (Marshall & Pierce, 2012). Smalleye stingrays observed off the southern coast of Mozambique seem to fit many of these requirements in that they are easy to approach and are regularly encountered on inshore reefs (Andrea D. Marshall, pers. comm., 2018). This study aimed to test the final assumption, that: individuals can be both reliably distinguished from one another and re-identified over short and long periods of time (Marshall & Pierce, 2012).

Research dive logs were combined with opportunistic photographs from local dive centers and recreational divers to provide additional information about the ecology and behavior of this elusive ray. Furthermore, we provide the first information on the range, abundance, habitat use, migratory capabilities including rate of movement, of this rare species in Mozambique.

## Materials & Methods

### Study sites

**Figure 1:**
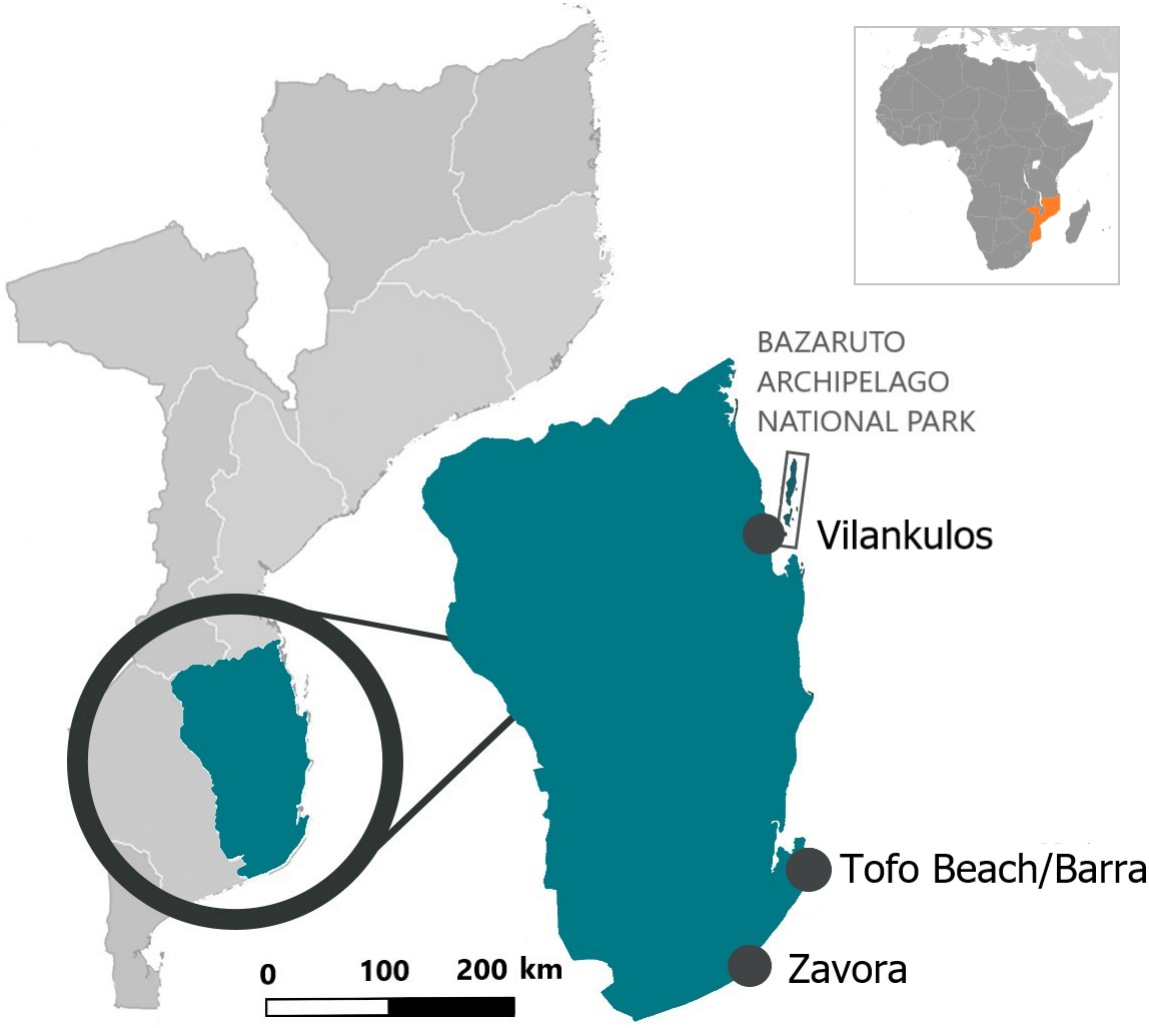
Location of the three study sites in the Inhambane Province coastline, Mozambique.

Opportunistic photographs of the dorsal surfaces of free-swimming stingrays were collected year-round by local researchers, dive operators and recreational divers in three main regions (Zavora, Tofo Beach/Barra, and the Bazaruto Archipelago National Park) of the Inhambane Province from May 2003 to November 2018 (Fig. 1). Opportunistic photographs were also taken of two individuals landed intentionally or as by-catch during the study period.

Tofo Beach (23°85′S, 35°54′E) and Barra (23°80′S, 35°52′E) lie on the Southern Mozambican coast, on Ponta da Barra peninsula in Inhambane Province. Tofo Beach/Barra dive sites are comprised mostly of rocky reefs with associated corals rising from the sandy seabed. Many of the reefs serve as year-round cleaning stations for elasmobranchs, most notably both species of manta rays *(Mobula alfredi* and *Mobula birostris*). The majority of the reefs can be found at depths between 22 and 32 m.

Zavora (24°23′S, 35°43′E) is located on the same coast, 80 km south west of Tofo Beach. Zavora dive sites are very similar to those found in Tofo Beach/Barra with the additional presence of kelp on the deep reefs. Reefs in this region also support year-round cleaning stations for elasmobranchs and are typically situated between 12 and 35 m of depth.

The Bazaruto Archipelago National Park (BANP) is a 1,583 km^2^ area encompassing five sand dune islands situated approximately 15 km from the coast, off the mainland city of Vilankulos (22°00′S, 35°32′E). Unlike the two other study sites, the Bazaruto Archipelago has more diverse tropical reef systems. Deeper reefs are similar to those in the south of the Province only with more coral cover, particularly soft corals. Shallow reefs comprise significantly more hard corals than in the south regions and the inside of the archipelago is mainly tidal sand flats with deep connecting channels to the ocean (South African Association for Marine Biological Research, 2008).

### Photographic identification

**Figure 2:**
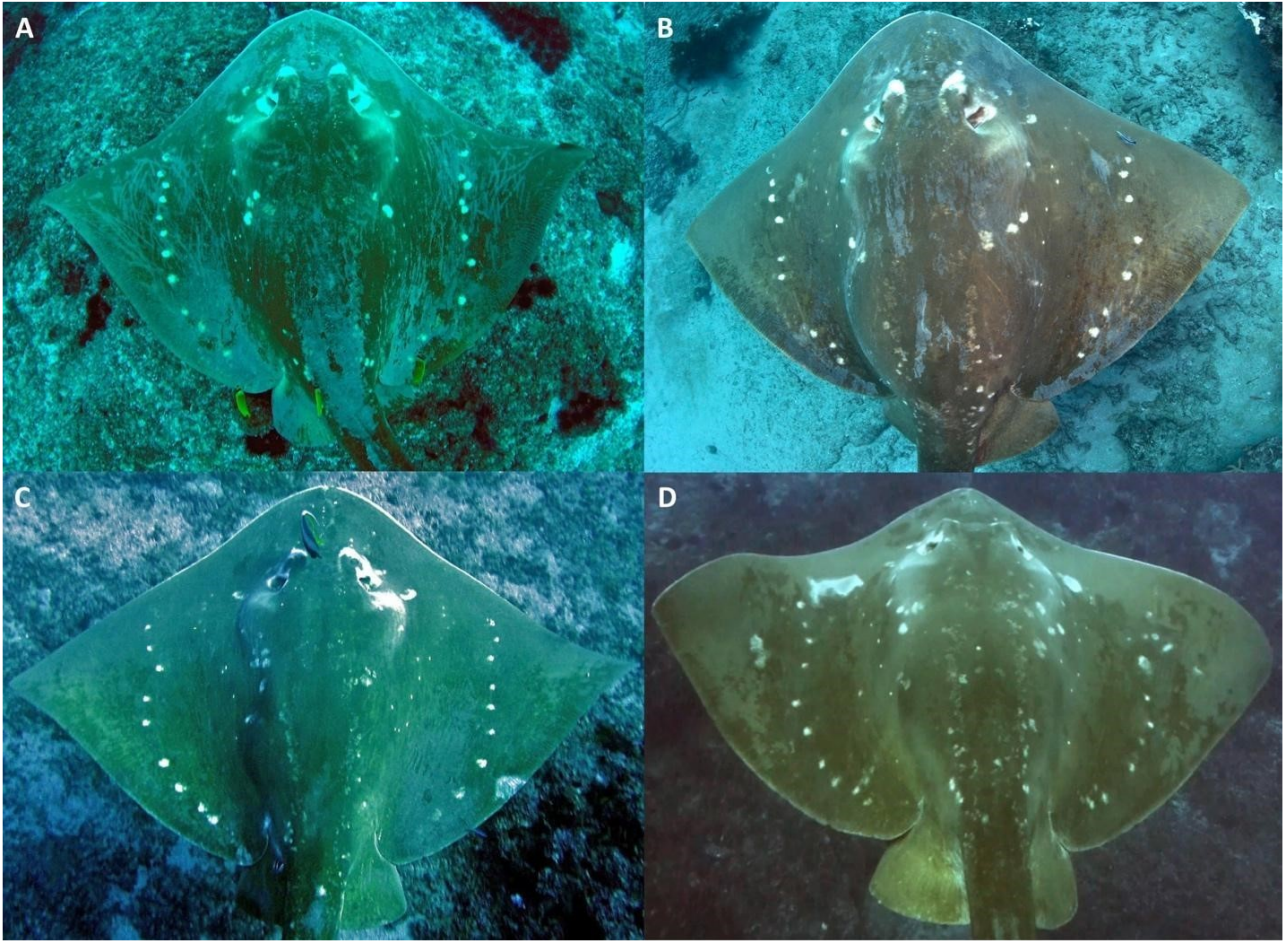
Variation in the natural spot patterns across the dorsal surface of *Megatrygon microps* from the Inhambane Province, Mozambique. ID-photos of (A) smalleye #070 off Tofo, September 27th 2007, (B) smalleye #052 in the BANP, May 6th 2017, (C) smalleye #023 off Tofo, May 4th 2013, and (D) smalleye #020 off Tofo, October 24th 2012. Photo credit: (A) & (B) Andrea Marshall; (C) Tom Horton; (D) Libby Bowles.

*M. microps* are medium-brown in colour, with a longitudinal row of large white spots on either side of the disc at about two-thirds distance from the dorsal midline to pectoral-fin apex (Fig. 2). Additional large white spots are lateral to the eyes and on either side of the mid-disc. Several rows of small white spots are located on either side of the base of the tail and of the disc, at about one third distance from the dorsal midline to pectoral-fin apex (Pierce, White & Marshall, 2008).

**Figure 3:**
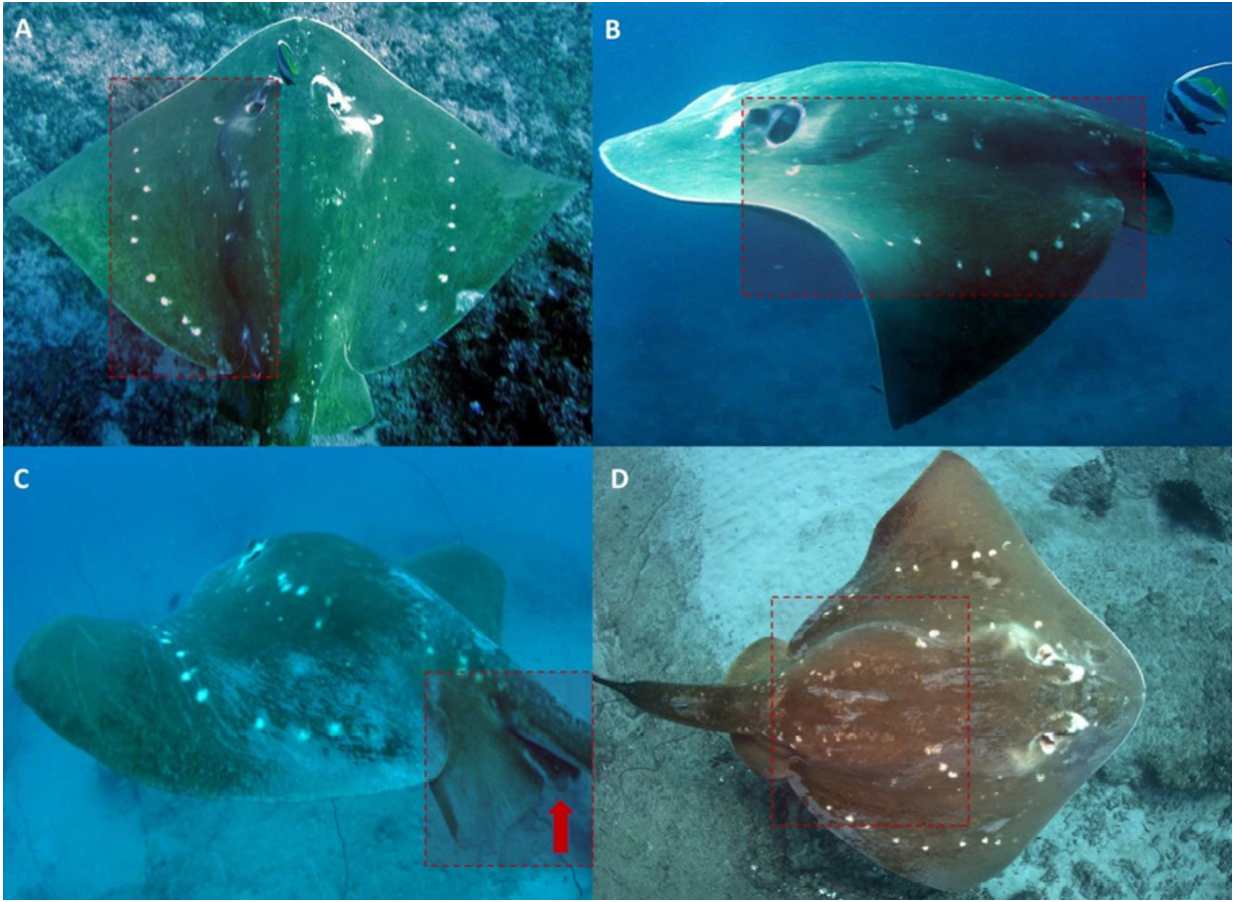
Standardized ID-area and attributes observed on encountered smalleye stingrays. Full dorsal (A) and partial (B) ID-photos of smalleye #023. (C) Males’ claspers are often obscured by the pelvic fins and the tail. (D) Distended abdomen of smalleye #052 suggesting gestation. Photo credit: (A) & (B) Tom Horton; (C) & (D) Andrea Marshall.

In order to investigate the variability of these natural spot patterns and monitor the longevity of markings, an image of the entire dorsal surface of the animal was attempted at every encounter. This region became the standardized identification area (ID-area), as the use of the entire spot patterns area across the flattened dorsal plane allows for efficient visual comparison between individuals with a higher degree of confidence than any one region alone (Fig. 3A).

Photographs of this standardized ID region (ID-photos) were taken from still photographs, with or without flash, or from video screenshots, with or without video lights. Only photographs of good quality were used for this study (147 out of 178 photographs in the study set). These images were in focus, clearly showed the natural spot patterns of the ray, e.g., was not obscured by sediment or associating fish, and had enough resolution to clearly distinguish the details of the spot patterns. However, photographs with only one side of the ID-area visible (partial ID-photos) were still usable for re-sightings: if a partial ID-photo was matched to an initial ID-photo with at least 10 spots clearly visible, the partial ID-photo was accepted as a re-sighting (Figs. 3A and 3B). Once appropriate ID-photos were secured, the sex of the animal was determined through the presence or absence of male reproductive organs (claspers) located on the pelvic fins (Fig. 3C). Scars, bite marks and deformities were also noted to provide a useful secondary verification of an ID, thus bolstering confidence in the accuracy of this validation study.

Matching was performed by visual pairwise comparison (two pictures side by side on the same screen), by one or two researchers. Each photograph was compared at least twice to the entire photo-database. Photographs were matched visually with no ambiguity, e.g., all visible natural markings were matched prior to confirming a re-sighting.

**Figure 4:**
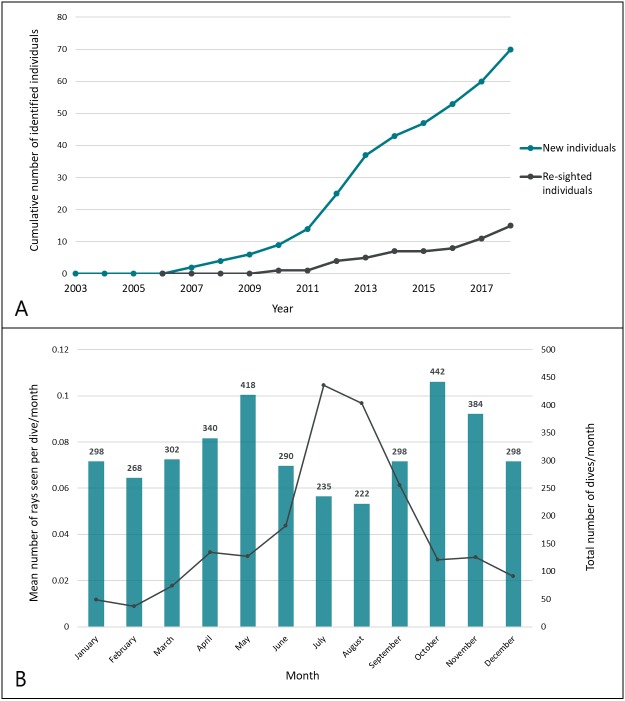
Recruitment of smalleye stingrays into the population database during the study period. (A) Discovery curves of new and re-sighted identified rays; (B) seasonal trends of encounters. The mean SPUE (number of sightings per dive) was computed for each month across all years of the study period. Sampling effort is indicated by the total number of dives/month.

New identifications were cumulatively plotted over time in a discovery curve to show the rate at which newly identified individuals were recruited into the population database (Fig. 4A). Re-sighting events were characterized by the positive identification of a previously identified individual more than 24 h after it was initially seen (e.g., not on consecutive dives) or at a different dive site. Rate of movement was calculated as the minimum time required to travel from one known location to another as confirmed by positive identification of individuals.

Re-sighting events allowed us to reveal some philopatric behaviors. Philopatry is defined as individuals frequently returning to or staying within their home ranges, birthplaces, or other specific localities (Chapman et al., 2015; Flowers et al., 2016). Two types of philopatric behavior were observed in this study: site fidelity, e.g., when the individual makes a long-distance movement away from a defined location and then returns to it (Chapman et al., 2015), and site affinity, e.g., when there is no available evidence to distinguish between site fidelity and residency, e.g., individuals remaining in a defined location for at least 12 months (Couturier et al., 2011).

To examine seasonal trends, the sightings per unit of effort (SPUE), e.g., the number of rays seen per dive and per month, was calculated for each year of the study period and then pooled by calendar month across all years. The means were compared across calendar months (Fig. 4B). Bottom Sea Temperature (BST) was recorded on each research dive with dive computers.

### Ethics statement

No approval or permits regarding human or animal ethics were required for this study as this was an analysis of publicly contributed photographs, collected non-intrusively, from recreational scuba divers. All data/photographs collected by authors were collected with special permissions from Bazaruto Archipelago National Park by the ANAC (Administração Nacional de Áreas de Conservação) of Mozambique and the Maritime Administration of Inhambane province.

## Results

*M. microps* dorsal spot patterns showed sufficiently unique variability to allow individual identification without ambiguity (Fig. 2). Natural spot patterns also remained unchanged in all individuals re-sighted during the study period with a maximum re-sighting interval of 2,214 days (6 years and 23 days), e.g., the maximum time interval between two sightings of the same stingray. The unique spot patterns, being quite distinctive, were easy to match by eye and did not require the use of pattern matching algorithms to confidently determine re-sightings.

Using these natural spot patterns, a total of 70 individual *M. microps* were identified from 182 sightings between May 2003 and November 2018 (Table 1), including 23 females, 17 males and 30 individuals of undetermined sex. Determining the sex of an individual during an encounter was secondary to obtaining an ID. Because the pelvic fin region was often obscured by the tail in ID-photos or difficult to view given the animal’s relative positioning to the diver, a high proportion of individuals could not be sexed (Fig. 3C).

**Table 1:** The survey effort in Zavora, Tofo Beach/Barra and Bazaruto Archipelago-San Sebastian areas, including sighting records and number of smalleyes identified. Main data come from research dives while bracketed numbers are the sightings provided by citizen contributions. SPUE (sightings per unit of effort) refer to the number of sightings of *M. microps* per dive, using research data only. Individuals encountered on different sites were counted in the site of the first encounter.

**Year**	**Zavora**			**Tofo Beach/Barra**			**Bazaruto Archipelago—San Sebastian**			**Total**		
	Sightings	Dives	SPUE	Sightings	Dives	SPUE	Sightings	Dives	SPUE	Sightings	Dives	SPUE
2003	0	0	–	0	33	0.00	0	0	–	0	33	0.00
2004	0	0	–	1	96	0.01	0	0	–	1	96	0.01
2005	0	0	–	1	94	0.01	0	0	–	1	94	0.01
2006	0	0	–	1	91	0.01	0	0	–	1	91	0.01
2007	0	0	–	7	125	0.06	0	0	–	7	125	0.06
2008	0	0	–	2	50	0.04	0	0	–	2	50	0.04
2009	0	0	–	1 (2)	64	0.02	0	0	–	1 (2)	64	0.02
2010	0	14	0	6 (1)	162	0.04	0	0	–	6 (1)	176	0.03
2011	0 (4)	0	–	13	351	0.04	0	0	–	13 (4)	351	0.05
2012	0	0	–	17 (5)	380	0.04	0	1	0.00	17 (5)	381	0.04
2013	2	28	0.07	20	266	0.08	3	60	0.05	25	354	0.07
2014	0	11	0.00	16	299	0.05	3	163	0.02	19	473	0.04
2015	0	2	0.00	6 (3)	211	0.01	0	42	0.00	6 (3)	255	0.02
2016	0 (1)	2	0.00	17	358	0.05	0	4	0.00	17 (1)	364	0.05
2017	0 (2)	25	0.00	18 (2)	378	0.05	1 (1)	35	0.03	19 (5)	438	0.05
2018	0 (3)	5	0.00	20 (3)	432	0.05	0	13	0.00	20 (6)	450	0.06
*Total*	2 (10)	87	0.02	146 (16)	3390	0.04	7 (1)	318	0.02	155 (27)	3795	0.04
***M. microps identified (N)***	5			60			5			70		

As expected, the majority of encounters and thus identified individuals were from the Tofo Beach/Barra region, where dive and monitoring efforts are significantly higher than other locations. All smalleye stingray encounters in this region occurred on reefs between 15 and 30 m, except for two individuals which were spotted just below the surface (2–4 m). In Zavora, smalleye stingrays were encountered on both shallow (12–24 m) and deep reefs (25–35 m). In the Bazaruto region, smalleye stingrays were observed both inside and outside the park with sightings concentrated on two deep reefs (26–36 m), both of which are surrounded by large flat sandy areas. Notably these reefs both host cleaning stations regularly used by manta (South African Association for Marine Biological Research, 2008) and other ray species (Murie & Marshall, 2016). We recorded two fished smalleyes in Tofo (2006) and Bazaruto (2013) areas but were unable to identify either with our ID photo catalogue.

New individuals were steadily identified throughout the study period (Fig. 4A), with 70 individual *M. microps* ultimately identified between 2006 and 2018. Sightings occurred throughout the year in waters ranging between 18 and 28 °C. Peak encounters occurred between May and October (winter in southern Mozambique) and the most sightings per unit effort occurred in July with an average of 0.10 individuals sighted per dive (Fig. 4B).

Encountered *M. microps* individuals were always actively swimming and were never encountered resting on the seabed. Stingrays were generally solitary. Occasionally (18 dives during the study period) two or three individuals were sighted on the same dive and in some cases (8 dives during the study period) were seen swimming together in the same direction in apparent association. Encountered stingrays were often spotted at cleaning stations where small fish such as *Heniochus acuminatus* appeared to be removing parasites and other build-up from the ray’s skin (Fig. 3B). No foraging behaviour was observed during the study period.

**Figure 5:**
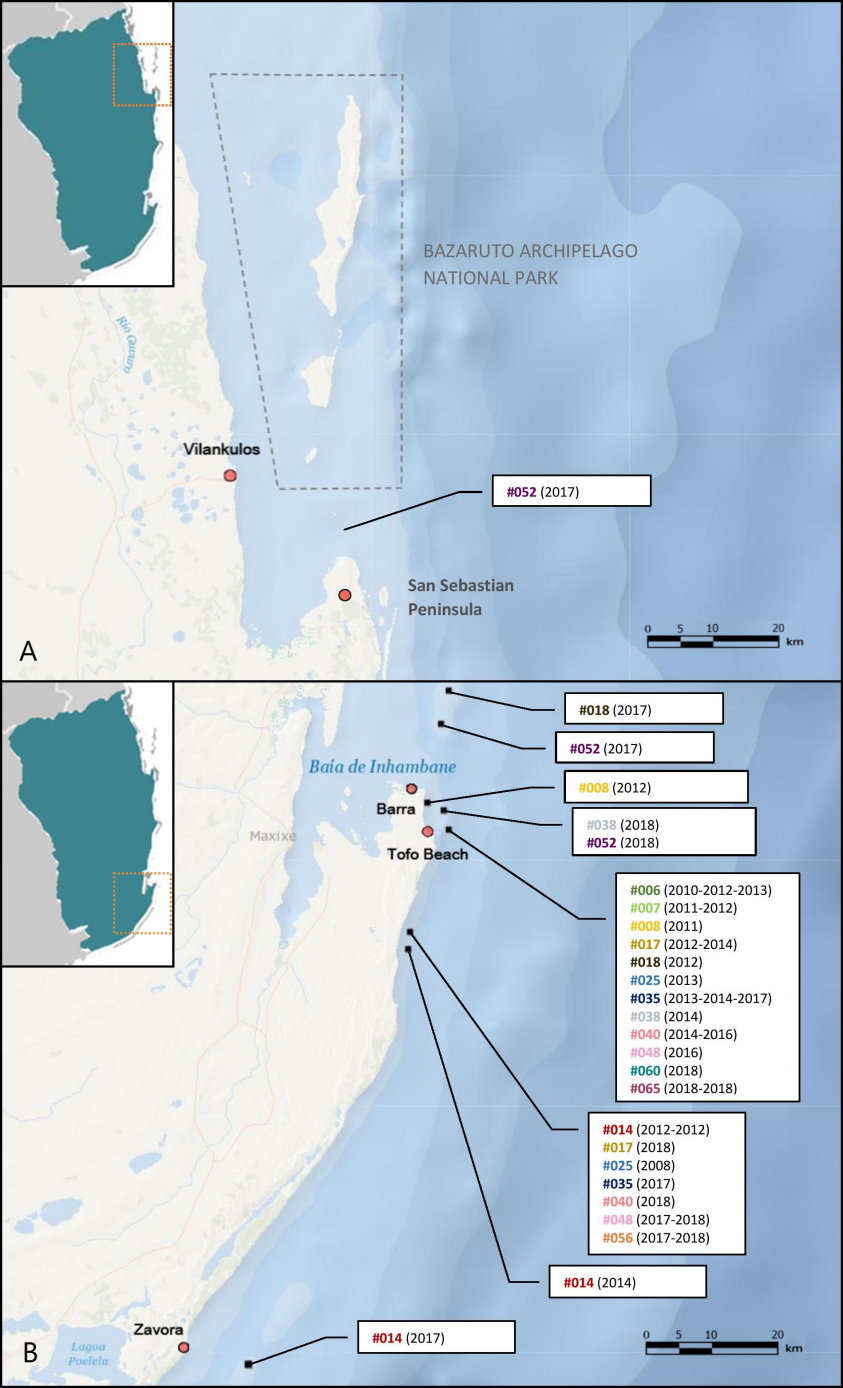
*M. microps* re-sighting records between 2003 and 2018 along Inhambane Province coastline. Encounters (A) in the Bazaruto Archipelago National Park region and (B) in Tofo Beach/Barra and Zavora regions. Bracketed numbers are the years of sightings for each individual-dive site pair: “#014 (2012-2012)” means that the smalleye #014 was spotted twice on this dive site in 2012. Base map credits: NIWA, Esri, DeLorme, NaturalVue.

**Figure 6:**
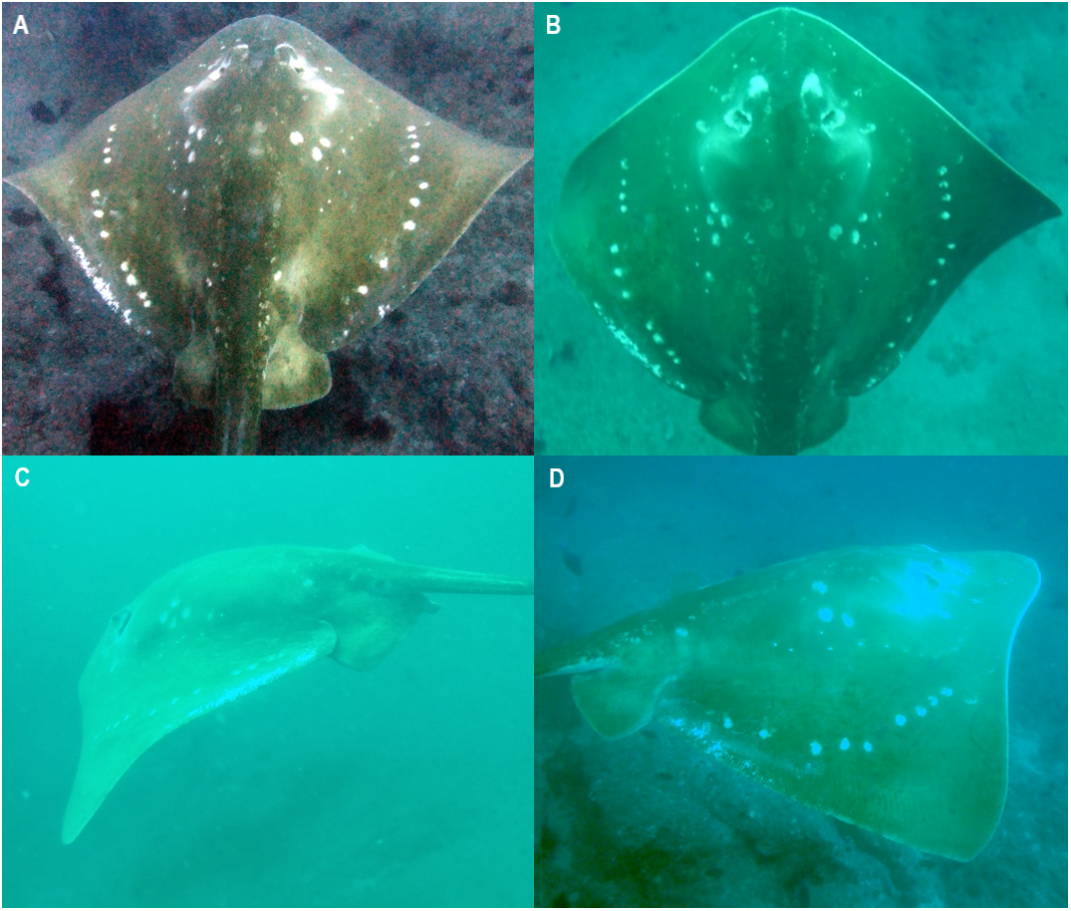
Smalleye #017, female encountered three times off Tofo, between 2012 and 2018. (A) July 21st 2012, (B) August 12th 2014, (C) and (D) August 13th 2018. Photo credit: (A) anonymous diver; (B) Libby Bowles; (C) & (D) Leslie Roberson.

A total of 15 of the 70 individuals identified (21.4%) were sighted more than once. Re-sighted individuals were seen on average 2.7 times during the study period (Fig. 5). The shortest time interval between a re-sighting of the same individual was 1 day, and the longest re-sighting occurred 2,214 days (6 years and 23 days) after the first sighting (Fig. 6). Most of the re-sightings occurred in Tofo Beach/Barra area, but this may be an artefact of greater effort in this region. Some of the re-sighted stingrays showed site affinity, with seven individuals observed on the same dive site over several years.

**Figure 7:**
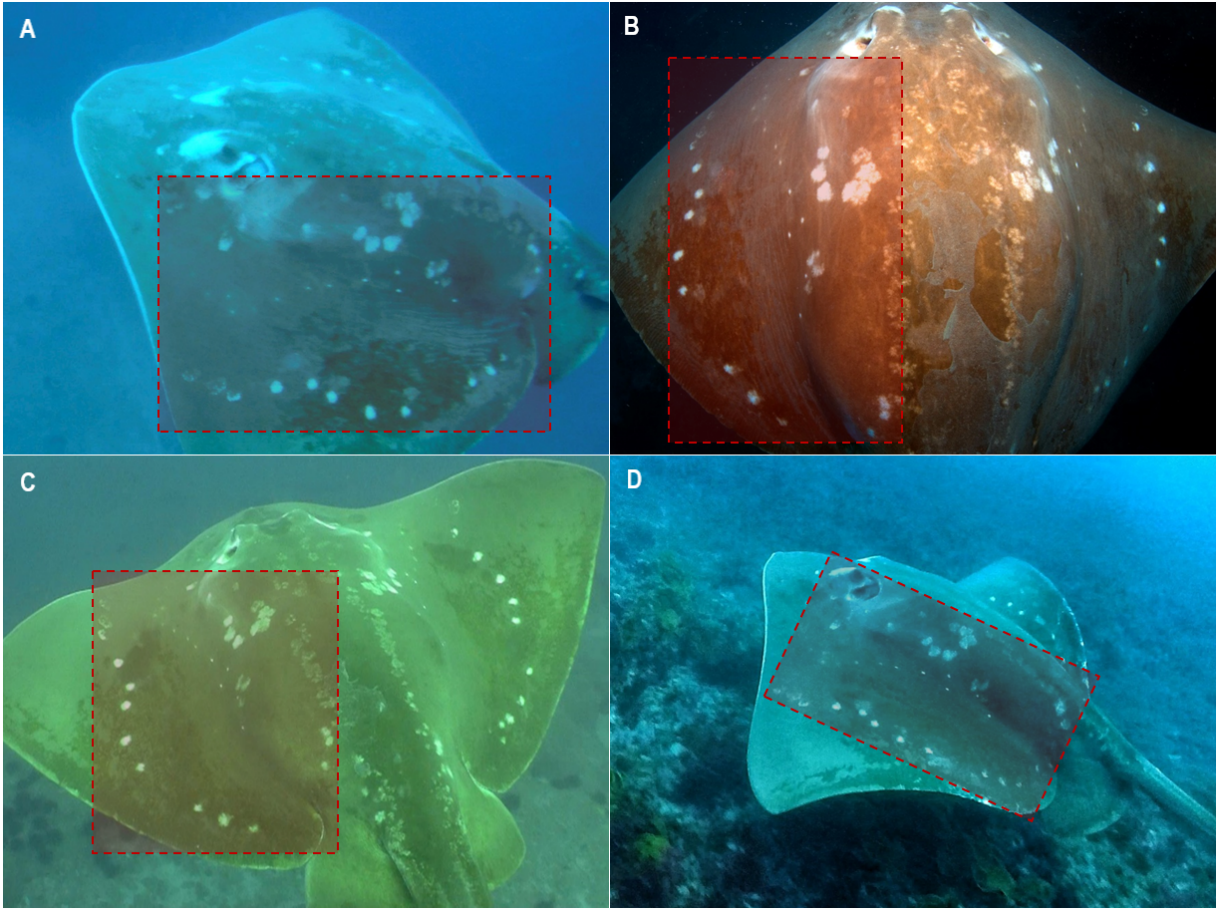
Smalleye #014, female encountered several times off Tofo. (A) March 21st 2012, (B) March 22nd 2012, (C) September 28th 2014. The last encounter occurred off Zavora on November 22nd 2017 (D). Photo credit: (A) Andrea Marshall; (B) Giles Winstanley; (C) Anna Flam; (D) Nakia Cullain.

**Figure 8:**
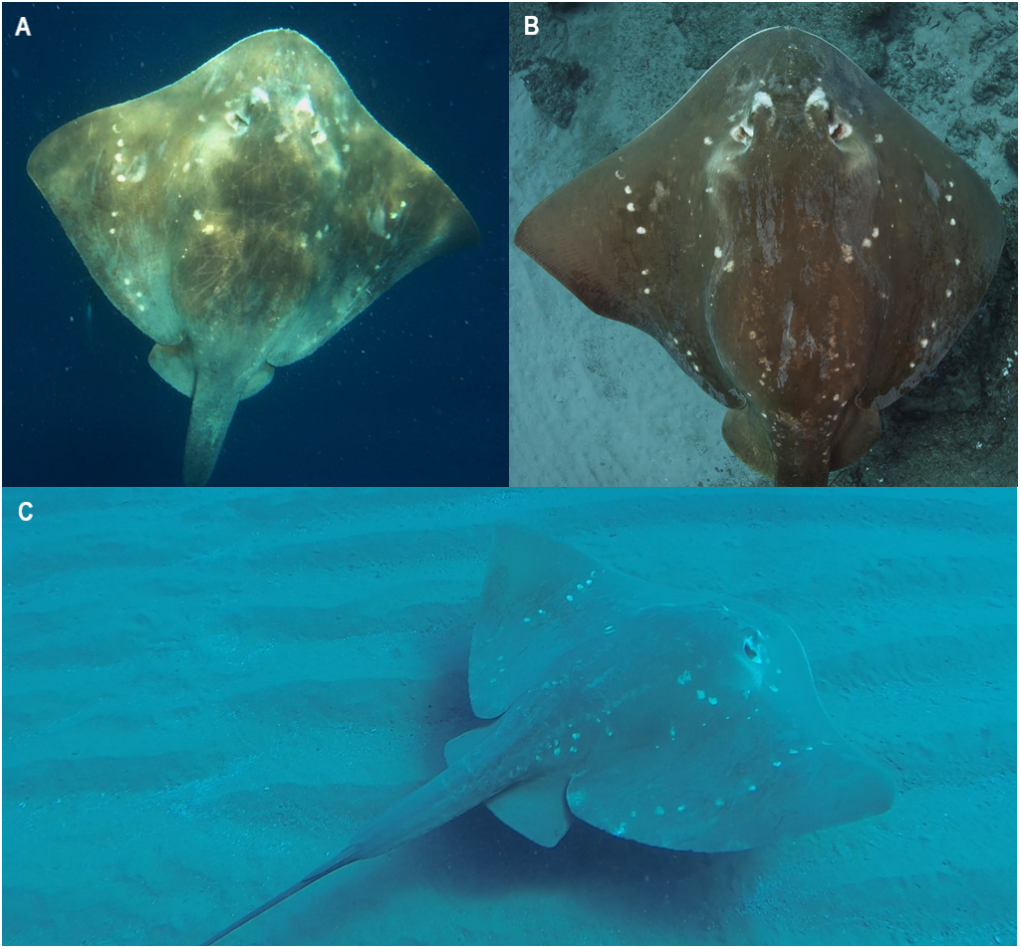
Smalleye #052, female encountered in Tofo Beach/Barra and Bazaruto areas and showing a return movement between both sites. (A) January 24th, 2017, off Tofo; (B) May 6th, 2017, near San Sebastian Point; (C) July 13th, 2018, off Tofo. Photo credit: (A) Michelle Carpenter; (B) Andrea Marshall; (C) Tyler Davis.

Re-sighting data also revealed inter-site movements between Zavora, Tofo Beach/Barra and the Bazaruto Archipelago. One individual was seen twice off Tofo Beach in 2012 and 2014, and then was subsequently re-sighted off Zavora in 2017, a minimum straight-line distance of approximately 70 km traveled by the ray within 1151 days (Fig. 7). Female rays, which appeared pregnant due to their distended stomachs and backs (Fig. 3D), were sighted in all three of the main study sites. One female identified off Tofo Beach on the 24th of January 2017 was re-sighted just south of the BANP, near San Sebastian Point, having traveled a minimum straight-line distance of approximately 200 km in a minimum of 102 days. The re-sighting of this stingray back in Tofo waters in June 2018 reveals clear site fidelity to this area (Fig. 8). The stingray was no longer visibly pregnant.

## Discussion

### The use of photo-ID for *M. microps*

True validation of photo-ID ideally requires double marking (e.g., conventional tagging and photo-ID) of the animals in order to have a reliable and independent confirmation of re-sighted individuals’ identities. Like in many terrestrial (Del Lama et al., 2011; Jackson et al., 2006) and marine studies using photo ID (Bansemer & Bennett, 2011; Domeier & Nasby-Lucas, 2007), this work did not include any conventional double marking techniques to validate patterns on individuals. However, enough data were presented to convincingly demonstrate the accuracy of matches, due namely to the clear and variable spot patterns on this species.

This study suggests adequate variability exists in the natural spot patterns on the dorsal surface of *M. microps* to reliably study wild populations with photo-ID methodology. These naturally occurring spot patterns were stable and unchanging over periods of up to six years, suggesting they are permanent and fixed, at least after maturity. Based on the stingrays’ disc width (>1m), it is likely that most if not all of the individuals encountered and identified during this study were mature. Further research is needed to confirm if this species undergoes ontogenetic changes in colouration like in many orectolobiform sharks (Dingerkus, 1986). Additional monitoring would be required to confirm the long-term stability of these natural markings over the lifespan of the individual, which is currently unknown. Given the variability and stability of these natural spot patterns and the ease of which they can be captured, we recommend that photo-ID methodology may be a good starting point for future studies on wild populations of *M. microps*.

Furthermore, photo-ID suitability of this species makes it a good candidate for citizen science contributions. Both videos and still images provided high quality ID-photos. Videos often offered different angles of the animals and their pectoral disc, which proved useful in obtaining the best angle for a high-quality screengrab. Footage and photographs should ideally be taken from above the ray to see the full ID-area. Divers should abide by the following code of conduct: approaching slowly, staying 2–3 m away from the ray, and not chasing the animal. In this particular region, due to the wide-ranging nature of the species, their broad distribution along the coastline and increasing number of identified individuals, future research may explore the feasibility and accuracy of pattern matching algorithms (e.g., I^3^S or Wildbook®). Those may help to facilitate the development of local/regional databases and the incorporation of citizen science contributions.

### Movement patterns and habitat usage

This study extends the known area of occurrence of *M. microps* in southern Mozambique and suggests that this species may be widespread in the south of the country. Opportunistic collection of photographs from divers helped establish connectivity along a 350 km stretch of coastline in the Inhambane Province, from Zavora in the south to Bazaruto Archipelago in the north. Individuals were found to travel long distances north and south along the coast over short and long periods of time, at least 200 kilometers in as little as 102 days, and 400 kilometers in 535 days which is the longest distance traveled by a dasyatid ray on record. The only similar numbers reported on a dasyatid ray’s movements range from 151 to 258 kilometers, distances traveled by four pelagic stingrays over the course of a 13-day satellite tag recording period (Weidner, Cotton & Kerstetter, 2014). The pelagic stingray however is likely to be more mobile than the smalleye stingray with suspected, but yet to be confirmed, seasonal migrations of hundreds to thousands of kilometers (Mollet, 2002)*.*

*M. microps*’ ability to travel long distances bolsters the supposition that the species is semi-pelagic (Pierce, White & Marshall, 2008), providing further evidence that it rarely seems to rest on the seabed like most stingrays. Like manta rays, another large and highly mobile species, *M. microps* also utilizes a wide variety of habitats along the coastline. Furthermore, like manta rays in Inhambane they are most frequently encountered on small deep reefs (20–35 m), particularly those that are surrounded by sand and support cleaning stations for elasmobranch species (Marshall, 2008). Similar to manta rays (Marshall, Dudgeon & Bennett, 2011), some individual *M. microps* show site affinity to these preferred reefs along the coast.

Both male and female rays were encountered during this study suggesting this species does not segregate by sex. However, no young of the year or even juvenile rays were observed suggesting that either ontogenetic shifts in resource or habitat use is driving the segregation of smaller sizes classes from the adults, which is common among other ray species (Marshall, Dudgeon & Bennett, 2011; Yick, Tracey & White, 2011; Espinoza et al., 2015). Individuals were most often encountered alone, either swimming through or cleaning at deep water reefs in the region, suggesting that cleaning may be an important factor to their visits to these reefs. Visits to cleaning stations have been shown to be linked with social behavior, including mating: solitary, highly mobile species like manta rays appear to use these reefs as meeting points where males can more easily locate and court receptive mates (Jaine et al., 2012; Stevens, 2016). Juvenile *M. microps* may also have smaller parasite loads and not need to clean as frequently as adults, or they may not be driven to visit these areas for social reasons until they reach maturity.

As with many dorsally flattened rays, late stage or near-term pregnancies were easily distinguishable (Marshall & Bennett, 2010) by distended bellies and particularly backs (Fig. 3D). Only one caught pregnant female smalleye stingray of 206 cm DW was previously examined off the East Indian coast and contained a single, late-term male embryo of 33 cm DW (Nair & Soundararajan, 1976). With visibly pregnant females sighted in all three of the study regions in southern Mozambique, it is likely that this area is regularly used by females to gestate young and parturition may occur in the immediate area. The observed long-distance movement of a near-term pregnant female from Tofo Beach-Barra to Bazaruto Archipelago suggests that *M. microps* may move into the warmer shallower waters of the archipelago to give birth. However, with no records of juvenile *M. microps* being landed in artisanal fisheries (Andrea D. Marshall, pers. comm., 2018), it is equally likely that the depth range of this species extends into deeper water than can be accessed by recreational divers and females may be using deeper waters as pupping grounds.

**Figure 9:**
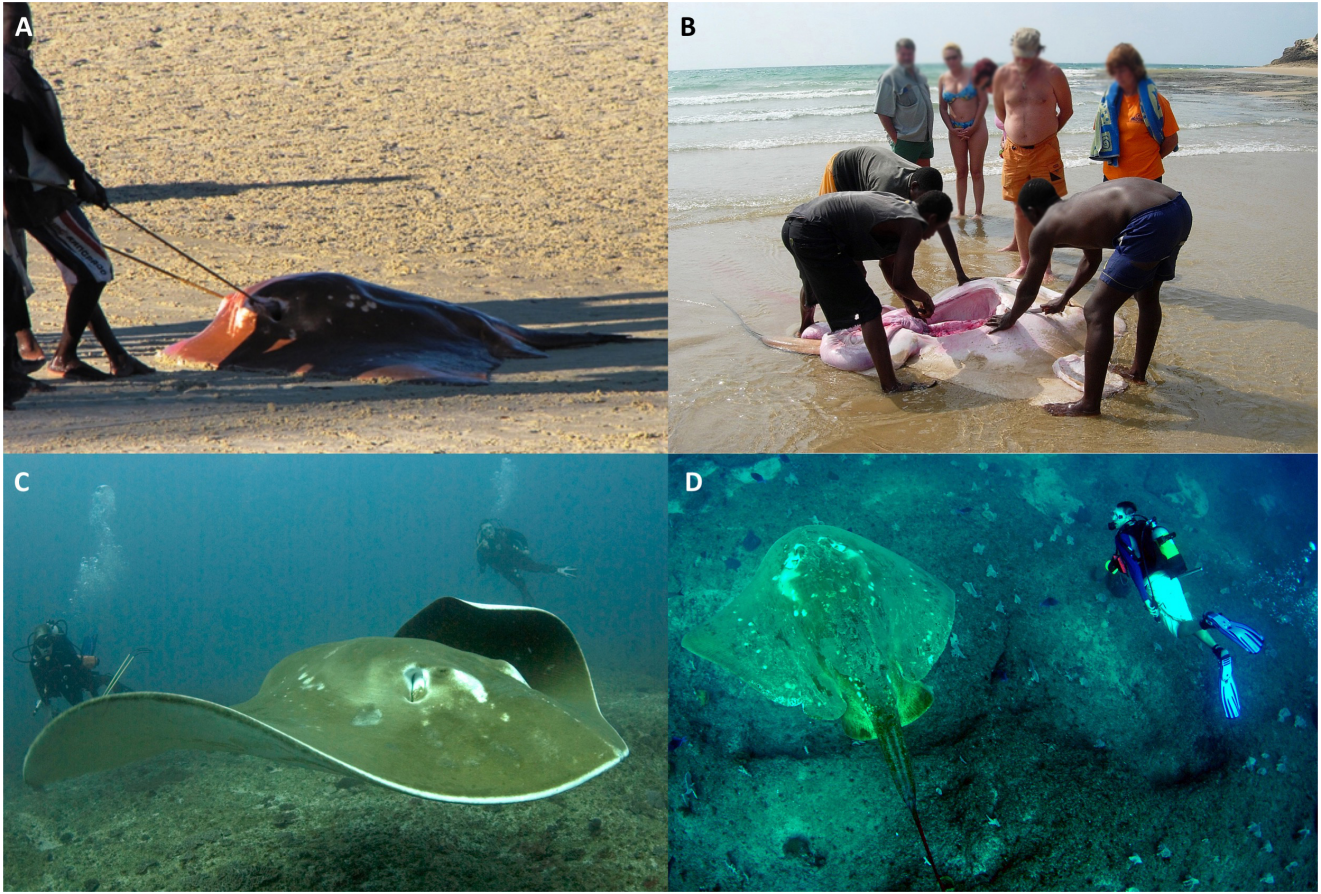
Human interactions with *M. microps* along southern Mozambican coastline. Individuals landed in artisanal fisheries on (A) Vilankulos Beach (May 27th, 2013) and (B) Tofo Beach (August 15th, 2006). (C) and (D) show smalleye stingrays encountered during recreational and research dives. Photo credit: Andrea Marshall.

While the discovery curve showed an increasing discovery rate since 2007, the relatively low sightings rate of this species indicates that it is rare, and that the population size off this coastline may be small. Increases in fishing pressure along this coast over the last decade have coincided with dramatic declines in other large ray species, like manta rays and devil rays (Rohner et al., 2013). *M. microps* are likely to be subject to similar pressures from targeted artisanal fishing and incidental capture in the gill and seine nets used extensively along the coast (Figs. 9A and 9B).

*M. microps* are large, charismatic, and easy to approach (Figs. 9C and 9D). As such, smalleye stingray encounters are highly sought after by divers (Steven Counsel, pers. comm., 2018). Like manta rays, which are attractions for diving tourism (Venables et al., 2016), it is likely that diving tourism is and will continue to be bolstered in this region by reliable encounters with this rare ray species. While the Bazaruto Archipelago National Park may offer a natural safe haven for these rays, it is recommended that management strategies be examined to increase protections of this lesser known, potentially threatened species throughout its range.

## Conclusion

Findings from the present study suggest that *M. microps* natural spot patterns are suitable for photo-ID methodology. Photo-identified smalleye stingrays in southern Mozambique proved to be using a wide variety of habitats and to be highly mobile along the coastline, with re-sighting events revealing migrations of up to 400 kilometers. Further investigation is required to validate the use of photo-ID on a longer-term basis. In the future, the information provided by this method will help in expanding ecological and demographic information on *M. microps*, fundamental for the overall knowledge of the species and needed to establish appropriate management and conservation strategies along Mozambican coasts.

##  Supplemental Information

10.7717/peerj.7110/supp-1 Data S1 Research dive logs and citizen contributed data The first two tabs show research dive logs and citizen scientist contributed data on encountered smalleye stingrays (raw data). The last two tabs provide sightings per year and region, and seasonal trends, based on research dive logs only.

10.7717/peerj.7110/supp-2 Table S1 Identified smalleye stingrays and their sightings over the study period Basic information collected on the 70 identified individuals were computed in this table: sex, partial/full ID-photos, dates of sightings.

## Additional Information and Declarations

### Field Study Permissions

The following information was supplied relating to field study approvals (i.e., approving body and any reference numbers): Field work and experiments were approved (i) in Inhambane province by the Maritime Administration of Inhambane province and (ii) in the Bazaruto Archipelago National Park by the ANAC (Administração Nacional de Áreas de Conservação) of Mozambique.

### Data Availability

The following information was supplied regarding data availability: Raw data is provided in the Supplemental Files.
